# Adaptation to stress in football athletes: The importance of cognitive appraisal

**DOI:** 10.3389/fpsyg.2022.939840

**Published:** 2022-08-24

**Authors:** A. Rui Gomes, Clara Simães, Catarina Morais, Ricardo Cunha

**Affiliations:** ^1^Psychology Research Centre, School of Psychology, University of Minho, Braga, Portugal; ^2^School of Nursing, University of Minho, Braga, Portugal; ^3^Health Sciences Research Unit: Nursing (UICISA: E), Nursing School of Coimbra (ESEnfC), Coimbra, Portugal; ^4^Research Centre for Human Development, Faculty of Education and Psychology, Universidade Católica Portuguesa, Porto, Portugal

**Keywords:** challenge perception, competitive stressors, emotions, competitive level, threat perception, stress, age, sporting level

## Abstract

This study analysed the adaptation of football athletes to competitive stressors regarding the upcoming match. For that, the study adopted a cross-sectional methodology using a critical incident approach. The participants were 352 young male football athletes, aged between 15 and 19 years (*M* = 16.91, SD = 0.99), who were competing in the national football championship. The results indicated that cognitive appraisal partially mediated the relationship between competitive stressors and emotions: athletes who perceived stressors as a challenge, tended to feel more control over the situation and more resourceful (coping perception), leading to a more positive emotional experience, while those perceiving the stressors as a threat were more prone to experience less control and more negative emotions. This mediation model was moderated by athletes’ competitive level (U17 or U19), as the role of challenge perception was more pronounced in the U19 team, while the relationship between threat perception and less control was only observed for the U17 team. In sum, the data reveals the importance of cognitive appraisal in young football athletes’ adaptation to competitive stressors, bolstering the theoretical models in this area and the importance of psychologists to consider these variables during intervention, particularly cognitive appraisal.

## Introduction

Stress is a prominent topic in daily life because of its impact on individuals’ living contexts, being well-known some harmful effect on physical and mental health (Cohen et al., 2007; Gomes, 2014; Monroe and Slavich, 2016). Stress can be understood as a set of negative reactions and feelings in response to adverse or demanding situations (Turner and Jones, 2014), depending on how one evaluates and deals with it, representing one of the main factors contributing to reduced sports performance (McGreary et al., 2020). In recent decades, a growing interest has been observed in the phenomenon of adaptation to stress in the sports context due to the demands placed on athletes who perform in top-level competitive sports that justify the stressful nature of this context (Barker et al., 2011; Gustafsson et al., 2017; Hase et al., 2019; McLoughlin et al., 2021). Although youth sport represents a positive contribution to physical and psychological health (Krustrup et al., 2010; Snyder et al., 2010; Strand et al., 2019), it has also been associated with negative effects, such as anxiety, fear of failure, reduced self-confidence, and burnout (Choi et al., 2014; Gustafsson et al., 2017). Thus, it is relevant to understand how young athletes adapt to stress in sports contexts and the role of psychological factors in how they evaluate and cope with potential stressors.

The study of adaptation to stress and underlying factors is well described by Lazarus (1991), Lazarus (1999) in the Transactional Stress Model, which suggested that the adaptation process should be analyzed considering cognitive, motivational, and relational factors. Specifically, the author argues that a comprehensive analysis of adaption to stress should consider simultaneously the *stressors* that trigger the situation, *how* the situation is evaluated by the individual (cognitive appraisal), and the feelings that *emerge* from the situation. In other words, there are four key variables involved in the adaptive process: the stressors, the cognitive appraisal of the stressful situation, the athletes’ ability to cope with stress, and the emotions arising from those situations. Regarding stressors, there is evidence that athletes face numerous stressors, as is the case of pressure to perform, social expectations, fear of opponents or injuries, fear of making mistakes, among others (cf. Arnold and Fletcher, 2012 for a review); however, it seems that it is not the presence of the stressors itself that leads to negative experiences being necessary to consider the processes of cognitive appraisal. According to the Transactional Model of Lazarus (1991), cognitive appraisal consists of two components: (1) primary cognitive appraisal, in which the individual attributes meaning to the stressful situation, checking whether it is according personal goals, values, and beliefs; and, (2) secondary cognitive appraisal, in which the individual analyses the personal resources to deal with the stressful situation.

Regarding the primary cognitive appraisal, which occurs once the athlete evaluates a situation as stressful, four types of appraisal can arise: (a) challenge, in which the athlete appraises the situation as stimulating and anticipates gains; (b) threat, in which the athlete anticipates that something negative may occur in the future; (c) benefit, in which the athlete identifies the situation as advantageous; and, (c) loss or damage, in which the athlete identifies the situation as harmful or damaging (Lazarus and Folkman, 1984). Next, the athlete will try to deal with the stressors by engaging in secondary cognitive appraisal and, therefore, analyzes: (a) the external *vs.* internal responsibility of the event; (b) coping in terms of personal resources to cope with the demands of the event; (c) control regarding the stressful event; and, (d) future expectations, that is the extent to which the situation can change for the better or worse, taking into account the athletes’ personal goals (Lazarus, 1991; Gomes, 2014). Taken together, it is possible to conclude that it is the cognitive appraisal of the situation, and not the situation itself, that determines the experience of stress (Dixon and Turner, 2018). This distinction of variables involved in cognitive appraisal has been studied in sports contexts. For example, Campbell and Jones (2002) found that stressors can be appraised in different ways (as challenging, threatening, harmful), depending on *how* athletes interpret the situation. Specifically, they demonstrated that athletes who perceive the stressor as a challenge also perceived more control over the situation; on the other hand, the stress levels were more severe when it was perceived as threatening or harmful.

The analysis of how athletes appraise the stressors allows for a deeper understanding of the stress experience (McGreary et al., 2020), which, in turn, can explain the subsequent impact on emotions and performance achieved by athletes. Some research that analyzed the relationship between emotions and performance in athletes (e.g., Lane et al., 2009, 2010; González-García and Martinent, 2020) suggested that positive emotional and psychological states, such as happiness, calm, and confidence, were associated with optimal performance (achievement of an important goal), whereas negative emotional and psychological states (e.g., anger and confusion) were associated with a dysfunctional performance and failure to achieve an important goal. These studies allow an understanding of the factors involved in the process of adaptation to stress; however, it is difficult to capture the whole experience of stress just by analyzing parts of the adaptation process, which reinforces the importance of capturing this process in an integrated way (Nicholls et al., 2012; Wong et al., 2015; Gomes, 2017). Thus, this study aims to provide an integrative framework to understand adaptation to stressors on young athletes by taking an integrative analysis of competitive stressors, cognitive appraisal of a stressful situation, and the arising emotions of the stressful situation, considering, at the same time, the athletes’ characteristics (i.e., competitive level) that can influence the cognitive processing of stressful situations and, consequently, the adaptation process.

### Stressors, cognitive appraisal, and emotions

The relations established between stressors, cognitive appraisal, and the emotions associated with the stressful experience have been considered by transactional (Lazarus, 1991) and interactive (Gomes, 2014) proposals of adaptation to stress. Specifically, this study analyses the relations between these variables in sequential conceptual logic. The first variable refers to competitive stressors organized in six dimensions (competitive readiness, performance, errors, social expectations, opponents, and injuries). The second variable was cognitive appraisal, which refers to how athletes interpret a stressful event. Cognitive appraisal is a pivotal variable in the adaptation to stress because negative reactions occur when the individual evaluates the external demands of a situation as exceeding their abilities and resources to cope with those same demands (Lazarus, 1991). Building upon Lazarus’ theoretical model, Gomes (2014) proposed the Interactive Model of Adaptation to Stress. This theoretical framework expands Lazarus’ Transactional Stress Model, specifically by arguing that an adaptation process will only begin if the individual considers the stressors as important for their personal goals. For this reason, athletes’ perception of the importance of the stressful situation (i.e., competitive match) was measured in this study to ensure that it only reported results from athletes who attributed some personal meaning to the situation they were involved in, so they could then assess primary (e.g., threat and challenge perceptions) and secondary (e.g., coping and control perceptions) cognitive appraisal of the competitive stressors.

The process of cognitive appraisal has consequences on athletes’ emotions, which represents the third variable in the analysis in this study. Emotions are understood as psychophysiological reactions resulting from the interaction with the environment, being inherently linked to cognitive appraisal (Lazarus, 2000; Tamminen et al., 2016). In fact, cognitive appraisal influences the quality and intensity of the emotions experienced during a stressful event, which in turn influences behavior and athletes’ performance (Lazarus and Folkman, 1991; Jones, 1995). In the sports context, there are indications that if the athlete appraises the situation as threatening or harmful, they will tend to experience negative emotions; while if the athlete appraises the situation as challenging or beneficial, they will tend to experience positive emotions (Nicholls et al., 2014; Meijen et al., 2019). In addition, the influence of the secondary cognitive appraisal needs to be considered since low future expectations and low coping perceptions may intensify negative emotions, whereas high future expectations and high coping perceptions may intensify positive emotions (Lazarus, 1991, 2000; Collins, 2019).

In sum, this study comprises three variables that are central in the understanding of human adaptation to stress, and their role in the analysis was conceptually determined: competitive stressors (i.e., sources of stress) were conceived as antecedent variables (predictor variable), the cognitive appraisal was as a mediator, and emotions were as a consequent variable (criterion variable). Thus, it is expected that:

*H1. The relationship between competitive stressors and emotions is mediated by primary and secondary cognitive appraisal. Specifically, it is expected a negative pattern of adaptation to stressors in this way: (a) higher perception of* stressors *predicts negative patterns of cognitive appraisal (lower challenge and higher threat perception, lower control, and coping), which, in turn, predicts a more negative emotional experience and a less positive emotional experience; and it is expected a positive pattern of adaptation to stressors in this way: (b) lower perception of stressors predicts positive patterns of cognitive appraisal (higher challenge and lower threat perceptions, higher control and coping), which, in turn, predicts a more positive and less negative emotional experience.*

### The moderating role of competitive level

Some studies indicate that the relationship between cognitive appraisal and emotions is not as linear and automatic as previously thought (e.g., Uphill and Jones, 2007; Nicholls et al., 2011). Thus, it is possible that other factors, such as the athletes’ personal attributes and sports characteristics, interfere in the relationship between stressors, cognitive appraisal, and emotions and can, consequently, influence the process of adaptation to stress. The Interactive Model of Adaptation to Stress (Gomes, 2014) refers to these aspects as antecedent factors and suggests that athletes’ characteristics (such as gender, age, competitive level, etc.) can influence how individuals appraise stressors and, consequently, can have an impact on their adaptation to stress. The author proposed that athletes’ characteristics should be analyzed as possible moderating variables of the relationship between stressors and the outcome of stress adaptation.

Indeed, previous literature has found significant age differences in how athletes perceive and deal with stressful events in sports contexts. For example, Martens et al. (1990) wrote a literature review in which they concluded that competitive trait anxiety is age-related, arguing that younger children, when compared to older athletes, experience less anxiety regarding their participation in casual sports (such as playground matches) but more trait anxiety in structured sports settings. According to the authors, this is due to the awareness of the competitive nature of these situations (not salient in the playground, but very explicit when playing a formal competition). However, there are also indications that younger athletes report higher sport-related anxiety than their older teammates (e.g., Wong and Bridges, 1994). Reeves et al. (2009) provided additional indications suggesting that even though some stressors are similar across ages (e.g., making errors, performance), older adolescents show a greater number and wider range of coping strategies compared to younger adolescents. At the same time, older adolescents tend to use more problem- and emotion-focused strategies and fewer avoidance strategies when compared to younger adolescents (Goyen and Anshel, 1998; Reeves et al., 2009). These results are consistent with the ones found by Bebetsos and Antoniou (2003), who reported that older badminton athletes cope better with psychological distress and report higher emotional self-control. These results suggest that the age group needs to be considered and that older athletes may have gained, through experience, greater coping strategies and, therefore, are more proficient in dealing with stressful situations, such as sports competitions. In our study, we analyzed the role of age by collecting data from two competitive levels (athletes until 17 years old and athletes until 19 years old). This option allowed the division of athletes according to the competitive demands that are formally established by the respective national federation. Therefore, it is expected that:

*H2. The relationship between competitive stressors and emotions is mediated by primary and secondary cognitive appraisal (as stated in H1) and is moderated by athletes’ competitive level (U17 and U19). Specifically, it is expected that the negative pattern of adaptation to stressors predicted on H1 (lower challenge and higher threat perception, lower control and coping) is stronger in younger athletes (U17), and the positive pattern of adaptation to stressors (higher challenge and lower threat perceptions, higher control and coping) is stronger on older athletes (U19).*

**FIGURE 1:**
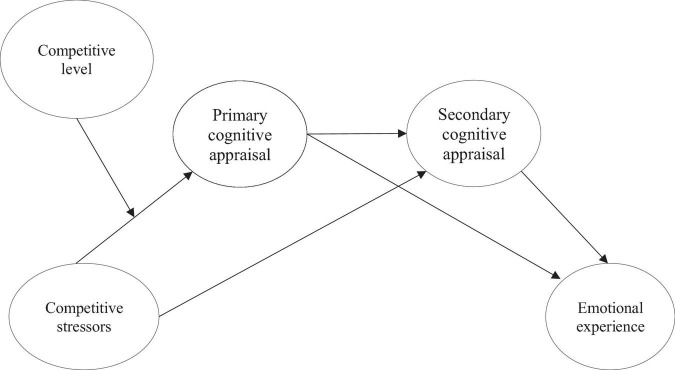
Proposed model of the study.

In sum, filling an important gap in the literature (Nicholls et al., 2012; Wong et al., 2015; Gomes, 2017) by pursuing a comprehensively investigating of the process of adaptation to competitive stressors, this study sought to analyze (1) the mediating role of cognitive appraisal in the relationship between competitive stressors and emotional experience; and (2) the moderating role of competitive level in the relationship between competitive stressors, cognitive appraisal, and emotions (cf. Figure 1). Based on the theoretical proposals formulated previously (cf. Lazarus, 1991, 1999; Gomes, 2014), we seek to understand how athletes adapt, evaluate, and react to a specific stressful situation in sport, in this case, the performance of an important match. Specifically, athletes completed the research protocol evaluating competitive stressors, cognitive appraisal, and emotions regarding an upcoming match. The variables of the study were organized according to the theoretical lines of the study of human adaptation to stress (Lazarus, 1991, 1999; Gomes, 2014), namely the factors that can trigger stress in athletes when they are exposed to a critical incident (antecedent variable); the processes of cognitive appraisal (mediator variables); and, the emotional reactions (consequent variables), controlling the influence of the competitive level in these relationships (moderator variable).

## Materials and methods

### Participants

The study involved 352 male young soccer athletes included in the Portuguese national championship of soccer, with ages for total sample ranging from 15 to 19 years old (*M* = 16.91, SD = 0.99). In terms of competitive level, 189 (54%) were in the U17 teams (ages between 16 and 17 years old) and 163 (46%) in the U19 team (ages between 18 and 19 years old). Regarding the number of collective titles won by the athletes, 268 (76%) reported at least one title, while 84 (24%) did not obtain any title. The mean of years of practice in official competitions was 9.14 (SD = 2.19), ranging from 1 to 15 years.

### Measures

#### Sources of stress

The Questionnaire of Competitive Stressors in Sport (QCSS; Faria and Gomes, 2018) was used to evaluate the potential sources of stress associated with the athletes’ performance. Specifically, athletes were asked to evaluate the level of stress caused by different competitive stressors regarding the upcoming match (*0* = *no stress, 4* = *very stressful*). The 24 statements were divided into six dimensions (4 items each): (a) competitive readiness: stress related to the athlete’s concern about not being well prepared for competing (e.g., “Not feeling ready for this next match,” α for this study = 0.77); (b) performance: stress related to athlete’s concern about having a bad performance or a performance below expectations (e.g., “To be defeated or have a bad result in this match,” α for this study = 0.80); (c) errors: stress related to the athlete’s concern about failing or making mistakes in important moments of competitions (e.g., “To fail in important moments of the match,” α for this study = 0.86); (d) social expectations: stress related to athlete’s concern related to not corresponding to what is expected of them and receiving negative evaluations from others (e.g., “Not match what others expect from me,” α for this study = 0.76); (e) opponents: stress related to athlete’s concern about competing with high qualified opponents (e.g., “To play against an opponent that is as good or better than me,” α for this study = 0.88); and (f) injuries: stress related to athlete’s concern about the possibility of getting injured (e.g., “Getting injured during the match,” α for this study = 0.73). The final score of perceived stress was computed through the average of the items’ scores of each dimension. Confirmatory factor analysis was performed to test construct validity and the instrument showed good psychometric properties in this study: χ^2^ (234) = 594.81, *p* < 0.001; χ^2^/*df* = 2.54; RMSEA = 0.066, C.I. [0.060;0.073]; SRMR = 0.073; CFI = 0.919; TLI = 0.904.

#### Cognitive appraisal

The Primary and Secondary Cognitive Appraisal Scale (PSCAS; Gomes and Teixeira, 2016) was used to evaluate the cognitive appraisal, which is based on Lazarus’s transactional model (Lazarus and Folkman, 1984; Lazarus, 1991, 1999), in the interactive perspective of adaptation to stress (Gomes, 2014), and in the model of stress and pressure at work (Karasek, 1979). The instrument consists of 15 items answered on a seven-point Likert scale (e.g., 0 = *Not at all*; 6 = *Very much*). It evaluates two dimensions: (a) the primary cognitive appraisal; and (b) the secondary cognitive appraisal. In the first section (primary cognitive appraisal), athletes indicate the importance and personal significance of the match in question, including three subscales (3 items each): (a) *importance perception*: indicates the importance attributed by the athlete to the upcoming match (e.g., “This match is important to me,” α for this study = 0.89); (b) *threat perception*: indicates the extent to which the athlete evaluates the upcoming match as disruptive and negative (e.g., “This match is disturbing to me,” α for this study = 0.79); and (c) *challenge perception*: indicates the extent to which the upcoming match is evaluated as stimulating and exciting by the athlete (e.g., “This match is exciting to me,” α for this study = 0.80). Regarding secondary cognitive appraisal, athletes evaluated the resources they believe they possess to deal with and solve the situation in question, across two dimensions: (a) *coping perception:* indicates the extent to which the athlete feels he/she has the resources to deal with the demands of the upcoming match (e.g., “I am able to deal and solve the demands of this match,” α for this study = 0.84); and (b) *control perception*: indicates the extent to which the athlete feels he/she has the power to decide about what to do in the upcoming match (e.g., “What happens in this match depends on me and my abilities,” α = for this study 0.79). The final score is computed through the mean of the items’ scores in each dimension, with higher values meaning higher scores in each dimension. Confirmatory factor analysis was performed to test construct validity and the instrument showed good psychometric properties in this study: χ^2^ (80) = 139.538, *p* < 0.000; χ^2^/*df* = 1.744; RMSEA = 0.046, C.I. [0.033;0.059]; SRMR = 0.044; CFI = 0.975; TLI = 0.968.

#### Emotions

The Sport Emotion Questionnaire [SEQ; translated and adapted from Jones et al. (2005) by Faria and Gomes (2018)] was used to access subjective feelings associated with emotions in sport in five dimensions: (a) anxiety (e.g., “I am worried about the next match,” α for this study = 0.76); (b) dejection (e.g., “I am unhappy about the next match,” α for this study = 0.92); (c) anger (e.g., “I am infuriated about the next match,” α for this study = 0.85); (d) excitement (e.g., “I am excited about the next match,” α for this study = 0.73); and, (e) happiness (e.g., “I am happy about the next match,” α for this study = 0.93). The first three dimensions assess negative emotional reactions and the last two positive emotional reactions. The instrument consists of 22 items answered on a five-point Likert scale (0 = *Not at all* to 4 = *Extremely*). Athletes fulfilled the instrument by thinking about their feelings at that specific moment regarding the upcoming match. The final score is obtained through the mean of the items’ scores of each dimension, with higher values meaning a greater emotional experience of the dimension in question. Confirmatory factor analysis was performed to test construct validity and the instrument showed good psychometric properties in this study: χ^2^ (178) = 406.185, *p* < 0.000; χ^2^/*df* = 2.282; RMSEA = 0.060, C.I. [0.053;0.068]; SRMR = 0.086; CFI = 0.952; TLI = 0.943.

### Procedure

The study was first submitted and approved by the Ethics Committee of the first authors’ University (SECSH-0162015). In this study, a convenience sample was used considering the following inclusion criteria: (a) language (Portuguese speakers), (b) athletes were in conditions to compete in the next upcoming match, and (c) gender (only male players were included), (d) competitive level (part of the U17 or U19 teams), and (e) competitive level (playing in the main division of the national league). Once the clubs accepted to be part of the study, athletes were contacted to participate. Because U17 and U19 teams were chosen, some athletes were underage (below 18 years old). In these cases, permission was first granted from their legal guardians. After legal guardians (when applicable) and athletes agreed to participate, the data collection was scheduled.

Data collection followed a critical incident approach (cf. Flanagan, 1973; Hettlage and Steinlin, 2006), which is a procedure known to facilitate the research of significant occurrences (i.e., critical incidents). Critical incidents are commonly defined as an observable activity that is sufficiently comprehensive to allow inferences and predictions to be made (Bitner et al., 1994). The main aim of this approach is to have a deeper understanding from the individual’s point of view (in this case, of the athlete) regarding the cognitive, affective, and behavioral elements entailed in the situation (Gremler, 2004). Even though this approach is more commonly used in qualitative research, it is also appropriate for quantitative methods to describe the nature of the events and their relationships with other variables (cf. Gremler, 2004). Therefore, every data collection was scheduled for 48–24 h before an important match. To ensure that athletes would consider the upcoming match “important,” data was collected during the final stages of the national championship (i.e., stages that define the teams’ final classifications) being also adopted other criteria associated with the values of importance perception of the PSCAS instrument, as explained below. Athletes filled the research protocol in the presence of a research team member.

Before answering the research protocol, athletes had to read the study’s instructions and goals and sign the informed consent forms. In the case of underage athletes, the informed consent was signed by their parents or coaches. Next, the athletes answered the evaluation protocol, consisting of the demographic information and the instruments explained below. The questionnaire was completed individually in an appropriate room made available by each sports club. It lasted between 15 and 25 min. Athletes’ participation was voluntary and anonymously, and the confidentiality of the collected data was ensured.

### Data analysis

The first step consisted of analyzing the importance perception dimension of the cognitive appraisal instrument. According to Gomes (2014), adaptation to stress implicates that athletes perceive the potentially stressful situation as important. To ensure it, athletes who scored 2 or less in this subscale were not included in the analysis as they attribute low personal relevance to the game they were about to play. Following this criterion, three participants were removed from the analyses (final *n* = 352). An online *a priori* sample size calculator for structural equation models was used to define the appropriate sample size for the proposed model (cf. Soper, 2022). A medium effect size of 0.3 and a desired statistical power level of 0.80 at the probability level of 0.05 was used as criteria. Therefore, to test the proposed model, a minimum of 221 participants was recommended.

Regarding the statistical assumptions to conduct Structural Equation Modeling, we checked for normality (cf. Kline, 2011) and multicollinearity (cf. Marôco, 2014). Regarding the normality assumptions, skewness and kurtosis were assessed. No severe deviations from normality were found (−1.31 > *sk* < 2.16; −0.59 > *ku* < 4.11). Correlations and VIF coefficients were used to assess for the multicollinearity assumption, and no indications of multicollinearity were found (−0.31 > *r* < 0.44; 1.00 > VIF < 1.34). Therefore, all assumptions were met.

This study’s first analysis aimed to assess athletes’ psychological experience before an important match. To do so, descriptive analyses were conducted to check for the relations among the variables (sources of stress, cognitive appraisal, and emotions).

Then, to test H1 (referring to the mediating role of cognitive appraisal in the relationship between competitive stressors and emotions), structural equation modeling was used in AMOS Software^®^. To ensure that the proposed partial mediation was the model that fit the data best, it was compared against the direct model (where direct relationships from competitive stressors and cognitive appraisal to emotions were included) and the alternative total mediation model (similar to the proposed model but without any direct effects from competitive stressors to emotions included). In order to simplify the models by decreasing the number of parameters to estimate (cf. Marôco, 2014), first-order latent variables were created for the cognitive appraisal (challenge, threat, control, and coping perceptions), and second-order latent variables were created for competitive stressors and for positive (excitement and happiness) and negative (anxiety, dejection, and anger) emotions. To assess the quality of the mediation models, the following criteria were used: (a) chi-square statistics (χ^2^); (b) Root mean square error of approximation (RMSEA; Steiger, 1990), considering an adequate fit when its values rely upon between 0.05 and 0.08 and a good fit when below 0.05 (cf. Arbuckle, 2008); (c) Standardized root mean square residual (SRMR) for which a good fit is achieved when below 0.10 (cf. Kline, 2011); (d) Tucker-Lewis index (TLI; Bentler and Bonett, 1980) and Comparative fit index (CFI; Bentler, 1990), for which values between 0.90 and 0.95 indicate an adequate fit and above 0.95 a good fit (cf. Bentler, 2007; Marôco, 2014). Bootstrap analyses were conducted to calculate the total and indirect effects of the mediation model.

Finally, to test H2, which analyzed the moderating role of the competitive level in the relationship between competitive stressors, cognitive appraisal, and emotions. Indirect effects and moderation analysis were conducted on AMOS Software^®^ using bootstrap technique and multigroup modeling (cf. Marôco, 2014), respectively. The same criteria for the mediation model were used to assess the model fit.

## Results

### Descriptive statistics

Table 1 summarizes the descriptive statistics of the study variables (stressors, cognitive appraisal, and emotions) and the correlations among them. Regarding the stressors, it can be concluded that performance and errors are the most prevalent, while opponents are the weaker source of stress for athletes. In cognitive appraisal, athletes seem to have a higher tendency to perceive the game more as a challenge than a threat and as having the necessary resources (control perception) to deal with it. Regarding the intensity of emotions, happiness and excitement seem to be the most prevalent compared to anxiety, dejection, and anger.

**TABLE 1:** Means (Standard deviations) and correlations among the study variables (*N* = 352).

	*M* (SD)	1	2	3	4	5	6	7	8	9	10	11	12	13	14	15
**Competitive stressors**																
1. Competitive readiness	1.93 (0.81)															
2. Performance	2.32 (0.75)	0.64**														
3. Errors	2.24 (0.36)	0.67**	0.70**													
4. Social expectations	1.70 (0.85)	0.62**	0.60**	0.65**												
5. Opponents	1.04 (0.78)	0.44**	0.30**	0.41**	0.61**											
6. Injuries	1.82 (0.88)	0.63**	0.53**	0.52**	0.48**	0.31**										
**Cognitive appraisal**																
7. Importance perception	5.22 (0.02)	0.09	0.24**	0.17**	0.09	–0.01	0.12**									
8. Threat perception	1.34 (1.28)	0.21**	0.15**	0.24**	0.27**	0.31**	0.17**	−0.21**								
9. Challenge perception	4.92 (1.15)	0.07	0.12*	0.12*	0.04	0.01	0.02	0.64**	−0.12*							
10. Coping perception	5.10 (0.77)	−0.13*	0.06	−0.14**	−0.18**	−0.24**	−0.11*	0.37**	−0.28**	0.27**						
11. Control perception	4.07 (1.11)	−0.12*	–0.02	–0.09	−0.11*	–0.03	–0.09	0.20**	–0.05	0.16**	0.42**					
**Emotions**																
12. Anxiety	1.16 (0.78)	0.25**	0.23**	0.32**	0.30**	0.31**	0.17**	0.08	0.34**	0.15**	−0.23**	–0.07				
13. Dejection	0.47 (0.87)	0.16**	0.10*	0.01	0.15**	0.01	0.12*	−0.20**	0.20**	−0.22**	−0.15**	–0.10	0.21**			
14. Anger	0.49 (0.81)	0.15**	0.12*	0.01	0.15**	0.13*	0.12*	−0.12*	0.21**	−0.13*	−0.14**	–0.05	0.32**	0.81**		
15. Excitement	2.86 (1.03)	0.00	0.11*	0.08	–0.06	−0.12*	–0.02	0.43**	−0.14**	0.46**	0.25**	0.17**	0.17**	−0.23**	−0.12*	
16. Happiness	2.69 (0.90)	–0.02	0.05	0.01	–0.06	–0.02	0.03	0.35**	−0.17**	0.34**	0.13*	0.17**	0.05	−0.59**	−0.47**	0.55**

*p < 0.05; **p < 0.01.

Regarding the correlations, all stressors are positively related to each other and with threat perceptions. Only performance and errors, as sources of competitive stress, are positively correlated with challenge perceptions. Most stressors are also negatively related to coping perception and positively to negative emotions (anxiety, dejection, anger). Competitive readiness and social expectations are negatively related to control perception. On the other hand, performance was positively related to excitement (positive emotion), and opponents were negatively related to the same positive emotions.

Concerning the relationship between cognitive appraisal and emotions, threat perception was positively related to negative emotions and negatively related to positive emotions. On the other hand, the more athletes perceived the competition to be a challenge, the more positive their emotions (excitement and happiness), the higher their anxiety, and the lower their negative emotions (dejection and anger). Coping and control perceptions were also positively related to positive emotions. The coping perception was also found to be negatively related to negative emotions.

### Stressors and emotions: The mediating role of cognitive appraisal

**FIGURE 2:**
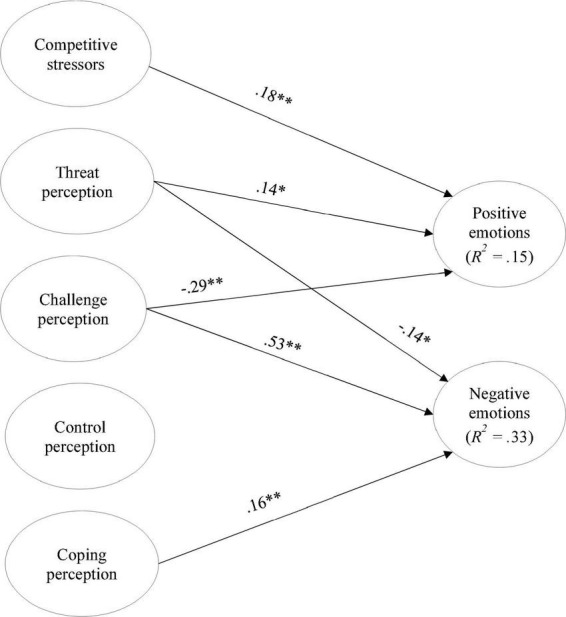
Direct model’s standardized coefficients. *p < 0.05; **p < 0.01; only significant paths are displayed.

**FIGURE 3:**
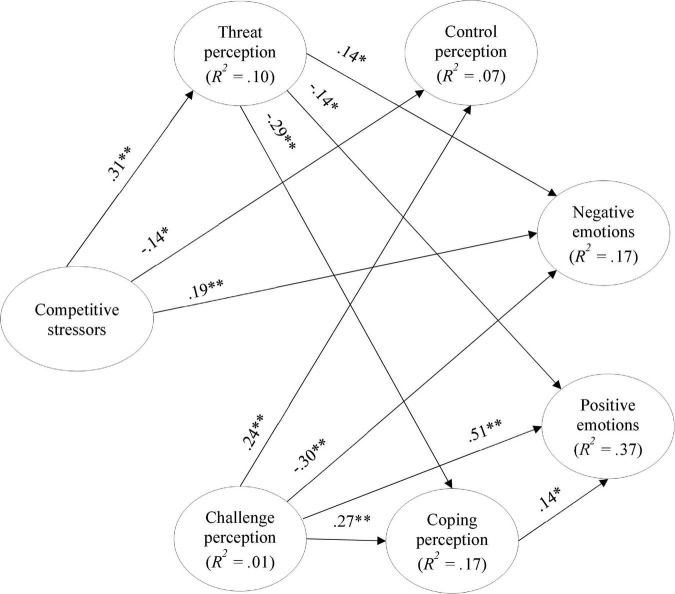
Standardized coefficients of the partial mediation model. *p < 0.05; **p < 0.01; only significant paths are displayed.

**FIGURE 4:**
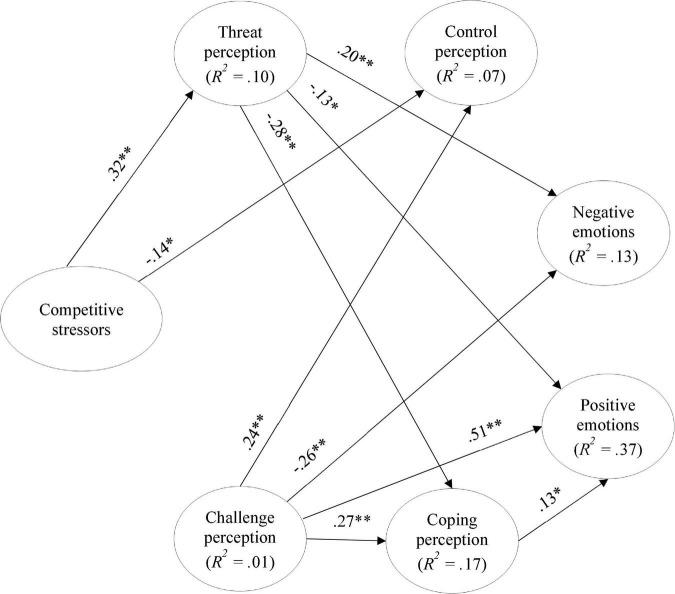
Standardized coefficients of the total mediation model. *p < 0.05; **p < 0.01; only significant paths are displayed.

To analyze the mediating role of cognitive appraisal in the relationship between competitive stressors and emotions, three different models were tested: (1) the direct model, which establishes a direct relationship between competitive stressors and cognitive appraisal of negative and positive emotions (cf. Figure 2); (2) the partial mediation model, including relations from competitive stressors to cognitive appraisal and emotions, but also direct relations from cognitive appraisal to emotions (cf. Figure 3); and (3) the total mediation model, which is similar to the previous model but no direct relations from competitive stressors to emotions were included (cf. Figure 4).

Table 2 summarizes the fit indexes obtained from the three models. The one that presents the best fit to the data is the partial mediation model. A chi-square test was conducted to assess the differences in the model fit. The results showed that the partial mediation model has a significantly better fit to the date than the total mediation model (Δχ^2^ = 9.92; Δ*df* = 2; *p* = 0.007), and then the direct model (Δχ^2^ = 97.88; Δ*df* = 8; *p* < 0.001). This result supported H1, meaning that the cognitive appraisal mediated the relationship between competitive stressors and emotions. According to the results, this mediation was partial.

**TABLE 2:** Fit indexes for the different models (*N* = 352).

Model	χ^2^	*Df*	χ^2^*/df*	RMSEA	RMSEA 95% CI	*p* (RMSEA)	SRMR	TLI	CFI
Direct	2542	1457	1.745	0.046	0.043; 0.049	0.986	0.107	0.895	0.904
Partial mediation	2444.12	1449	1.687	0.044	0.041; 0.047	0.999	0.092	0.903	0.912
Total mediation	2454.04	1451	1.691	0.044	0.041; 0.047	0.999	0.098	0.902	0.911

Thus, the partial mediation model’s coefficients were further analyzed. The model explained the variance of the perception of threat by 10%, challenge perception by 1%, control perception by 7%, and coping perception by 17%. Moreover, 17% of the negative emotions’ variance and 37% of positive emotions’ variance were explained by the predictor and mediator variables.

The direct effects (cf. Figure 3) showed that athletes who feel higher levels of competitive stressors also perceive higher threats about the upcoming match, experience lower perceptions of control, and stronger negative emotions. It was also found to have direct effects between perceiving higher threats and less coping and the experience of less positive emotions and more negative emotions. On the other hand, higher perceptions of challenges contribute to higher control and coping perceptions and more positive and less negative emotions. A positive direct effect between coping perception and positive emotions was also found.

The indirect and total effects are displayed in Table 3. The effect of primary cognitive appraisal on emotions was mediated by secondary cognitive appraisal. Specifically, higher threat levels predicted lower control and coping perceptions, which, in turn, lead to lower positive emotions. On the other hand, higher perceptions of challenges lead to higher perceptions of control and coping perception, which, in turn, predict more positive emotions.

**TABLE 3:** Indirect and total standardized effects (confidence intervals) of the partial mediation model.

	Secondary cognitive appraisal				Negative emotions		Positive emotions	
	Control perception		Coping perception					

	Indirect effect	Total effect	Indirect effect	Total effect	Indirect effect	Total effect	Indirect effect	Total effect
Competitive stressors	0.02	−0.12	−0.06	−0.17**	0.02	0.21**	−0.02	0.01
	[−0.051; 0.145]	[−0.461; 0.025]	[−0.117; 0.020]	[−0.265; −0.026]	[−0.041; 0.091]	[0.036; 0.279]	[−0.125; 0.079]	[−0.142; 0.166]
Threat perception		−0.03		−0.28**	0.02	0.16	−0.04*	−0.18*
		[−0.228; 0.092]		[−0.290; −0.085]	[−0.012; 0.043]	[−0.003; 0.193]	[−0.086; −0.001]	[−0.259; −0.026]
Challenge perception		0.24*		0.27**	−0.02	−0.32**	0.06*	0.57**
		[0.042; 0.482]		[0.061; 0.300]	[−0.053; 0.018]	[−0.252; −0.074]	[0.008; 0.107	[0.269; 0.523]
Control perception						−0.003		0.09
						[−0.064; 0.251]		[−0.064; 0.272]
Coping perception						−0.06		0.14
						[−0.213; 0.073]		[−0.019; 0.337]

*p < 0.050; **p < 0.010.

### Multigroup analysis: Model comparison amongst U17 and U19

This analysis aimed to compare the model with the best fit (partial mediation model) amongst U17 and U19 competitive levels. The central assumption was that the model would be invariant and that the model would behave similarly for both groups.

The first step consisted of comparing the invariance of the measurement model for the two groups; this was done by comparing the model with fixed weights against the unconstrained model (i.e., the model with all parameters free). Measurement model invariance would be achieved if *p*Δχ^2^ > 0.05 (Marôco, 2014) and or if ΔCFI < −0.01 (Cheung and Rensvold, 2002). Table 4 summarizes the multigroup analyses. Thus, assuming the unconstrained model to be correct, the results showed that the measurement model was different (i.e., not invariant) among the two groups (Δχ^2^ = 28.72, *p* = 0.011, as *p*Δχ^2^ < 0.05). However, the ΔCFI = −0.003, is a key criterion for the hypothesis of invariance of the measurement model not to be rejected because of the ΔCFI < −0.01.

**TABLE 4:** Comparative summary of fit indexes and multigroup invariance tests based on competitive level.

	χ^2^	*df*	*p*	χ^2^*/df*	TLI	CFI	RMSEA [90%CI]	SRMR	Δ χ^2^(a)	Δ *df*(a)	*p*(a)	Δ CF(a)	Δ χ^2^(b)	Δ *df* (b)	*p*(b)	Δ CFI(b)
**Total sample**																
Multigroup model	459.61	204	<0.001	2.253	0.914	0.931	0.06[0.05;0.07]	0.079								
**U17 *vs*. U19**																
Unconstrained model	697.01	408	<0.001	1.708	0.905	0.923	0.05[0.04;0.05]	0.087								
Measurement model	725.73	422	<0.001	1.720	0.904	0.920	0.05[0.04;0.05]	0.088	28.72	14	0.011	−0.003				
Structural model	762.60	440	<0.001	1.733	0.902	0.915	0.05[0.040;0.05]	0.096	65.58	32	<0.001	−0.008	36.87	18	0.005	−0.005

(a) Assuming the unconstrained model to be correct; (b) Assuming the measurement model to be correct.

**FIGURE 5:**
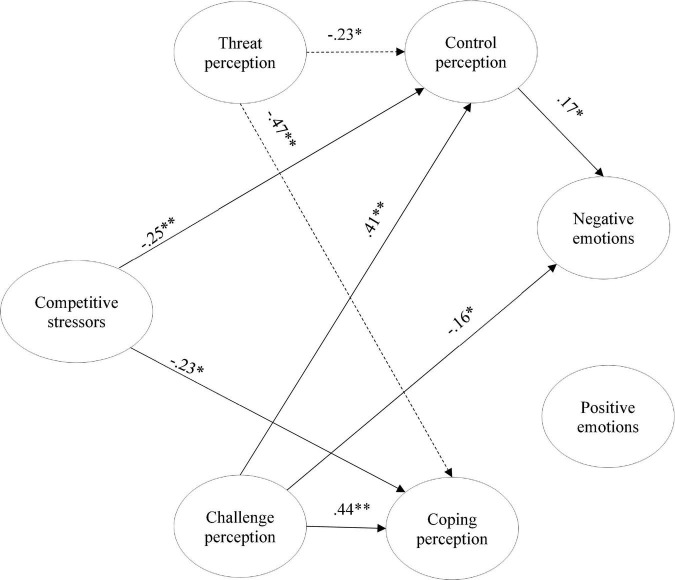
Partial mediation model: comparison among groups. The dashed arrows represent the U17, and the arrows the U19. *p < 0.05; **p < 0.01; only significantly different paths among groups are displayed.

Thus, assuming the invariance of the measurement model, the second step was to assess the invariance of the structural model. Therefore, assuming the measurement model to be correct, the structural model is different among the two groups (Δχ^2^ = 36.87, *p* = 0.005), which indicates that the competitive level acts as a moderator of the presented partial mediation model. This result supported H2, meaning that the relationship between competitive stressors and emotions was mediated by cognitive appraisal and moderated by athletes’ competitive level. The differences among groups are displayed in Figure 5. Results for the U17 athletes indicated that the negative relationship between threat perception and coping perception (*Z* = 3.04, *p* = 0.001) is significantly stronger; as is the case, the relationship between threat perception and control perception is significant (*Z* = 2.79, *p* = 0.003). Regarding the U19 group, two new relationships arise: higher perceived competitive stressors predicts lower coping perception (*Z* = −2.18, *p* = 0.015), and higher perceptions of control predicted more negative emotions (*Z* = 2.55, *p* = 0.005). When comparing to the U17 athletes, the relationship between perceived competitive stressors and perceptions of control (*Z* = −2.05, *p* = 0.020), and between challenge and control perceptions (*Z* = 2.64, *p* = 0.004), coping perception (*Z* = 2.02, *p* = 0.022), and negative emotions (*Z* = −1.94, *p* = 0.026) were stronger in the U19 group.

## Discussion

This study analyzed how young athletes evaluated and reacted to a particularly stressful situation (i.e., an important match to be played in the next 48 to 24 h). The relationship between competitive stressors, cognitive appraisal, and emotions was tested and compared among two competitive levels. The results indicated that performance and errors were important stressors. Regarding the mediation of cognitive appraisal between competitive stressors and emotions, two main aspects should be reinforced. First, higher perception of competitive stressors corresponded to higher experiences of negative emotions; in the same way, higher perception of competitive stressors corresponded to higher threat perception that, in turn, corresponded to less coping perception and lower experiences of positive emotions and higher experiences of negative emotions (confirming the negative pattern of adaptation to competitive stressors).

On the contrary, higher challenge perception corresponded to higher control perception and coping perception, higher experiences of positive emotions, and lower experiences of negative emotions (confirming the positive pattern of adaptation to competitive stressors). These results were found for the partial mediation model, confirming that the relationship between competitive stressors and emotions is mediated by the cognitive appraisal, confirming H1, although not all structural paths in the model were significant. It is also important to note that competitive level assumed a moderating role in these relationships among variables, confirming H2, although the structural paths are somewhat complex to interpret. Specifically, for U17 athletes, a negative relationship was found between threat perception and perceptions of coping and control, meaning that higher threat predicted less ability to cope and control the competitive stressors related to the upcoming match. For the U19 athletes, the set of significant relations increased, showing that a higher perception of competitive stressors predicted a lower perception of control (that, in turn, augmented the possibility of feeling negative emotions) and a lower perception of coping. Moreover, significant increases in challenge perception predicted higher control perception (in turn, augmented the possibility of feeling negative emotions).

Taking a closer look at the results and explicitly analyzing the stressors, we verified that that performance and errors were the highest sources of stress in sports, which is consistent with previous research (e.g., Noblet and Gifford, 2002; Sagar et al., 2010; Wong et al., 2015). Athletes’ fear of not performing according to the expectations and making errors may suggest some pressure felt by young athletes to be successful, which illustrates the least positive side of youth sport due to potential adverse effects (e.g., stress, anxiety, depression, burnout; Sagar et al., 2007, 2010; Gustafsson et al., 2017). Indeed, the global experience of competitive stressors was a predictor of athletes’ emotional experience. Specifically, athletes who experienced more stressors about the upcoming match perceived it as more threatening, as having less control over the outcome of the match, and felt more negative emotions. This fact is consistent with previous research (e.g., Nicholls et al., 2012; Gomes et al., 2013; Du et al., 2018), meaning that competitive stressors influence athletes’ emotional experience and how they cognitively evaluate the situation.

However, previous literature testing the Transactional Model of Stress also suggests that cognitive appraisal can help to mitigate these effects if athletes are able to interpret the situation as mainly challenging, which makes them cope positively with the sources of stress (Lazarus and Folkman, 1984; Dixon et al., 2020) and, as a result, experience more positive emotions (Palmwood and McBride, 2019). This idea is in line with the correlations and direct effects found in this study: challenge perception predicted higher experiences of positive emotions and lower experiences of negative emotions, which is aligned with previous research (e.g., Nicholls et al., 2011). Also, increases in challenge perception regarding the upcoming match corresponded to higher perceptions of secondary cognitive appraisal (coping and control perceptions). Taken together, the results of this study highlight the importance of considering cognitive appraisal when evaluating competitive stressors and their impact. Nevertheless, this adaptation to stressors may vary according to sport and personal characteristics, as suggested by the Interactive Model of Adaptation to Stress (Gomes, 2014). To test this assumption, in the present study, we analyzed the athletes’ competitive level, assuming that older athletes (U19) would have a stronger tendency to react more adaptively to stressors (i.e., tendency to perceive the match as challenging, more control and coping and, consequently, more positive emotions).

The results showed that the competitive level acted as a moderator, meaning that the partial mediation model presented some nuances based on athletes’ competitive level. Indeed, the U17 group, in comparison with the U19 group, assumed a more negative pattern of evaluating the competitive stressors by perceiving higher threat perception that decreased their perceptions of coping and control over the competitive stressors. The U19 group, in comparison with the U17, assumed a higher perception of competitive stressors that decreased both mechanisms of secondary cognitive appraisal; however, the U19 group seemed to mobilize a more challenging perception of competitive stressors that increased both the coping and control perceptions. In other words, the U17 seemed to evaluate the competitive stressors more threatfully, and the U19 seemed more aware of competitive stressors but evaluated the competitive stressors in a more challenging way (as expected). The increase of competitive stressors perception by U19 athletes can be related to an augment of competitive difficulty (which we could expect because they were competing at a higher competitive level), but they also seem to mobilize more challenge mechanisms. In contrast, the U17 athletes seem to be more preoccupied with negative aspects of competition which can deteriorate their mechanisms of coping and control. The role of competitive level in adaptation to stressors at younger ages is not easy to determine in literature. Previous studies provide evidence that the athletes’ age (and their respective competitive level in youth sports) are not related to psychological variables (González-García et al., 2020; Olsson et al., 2021), while other studies suggested some differences in the way athletes perceive and respond to sports stressors (Goyen and Anshel, 1998; Bebetsos and Antoniou, 2003; Reeves et al., 2009). Our results confirm these previous studies by reinforcing the need to consider the threat perception in younger athletes (U17 group), and the way athletes perceive competitive stressors in older athletes (U19 group).

### Limitations and future research

One limitation of this study is the cross-sectional design, which is an obstacle to inferring causality. However, it is essential to note that: (1) the proposed model has a strong theoretical basis, and (2) the use of a critical incident approach requires athletes to be questioned about a significant event at a very close time before the event. Indeed, athletes were questioned 48–24 h before the match, and (3) the importance of the event was ensured by using the final stages of the national championships, in which the teams’ ranking were still being decided. Nonetheless, it would be interesting to replicate the study with a longitudinal design to explore further the causal relationship between competitive stressors, cognitive appraisal, and emotional experiences. Also important, it would be useful to collect data besides self-reported measures from athletes by accumulating performance and skill execution indicators of athletes in competition and even in training sessions. The increase of time periods of evaluation aligned with a broader collecting of measures in distinct situations will provide a better understanding of how stressors, cognitive appraisal, and emotions interact to each other and explain how athletes adapt to sports demands.

Regarding the antecedent factors, literature suggests that athletes’ characteristics (such as gender or age, for example) may influence the adaptation process (e.g., Gomes, 2014). This study was conducted with male athletes because collecting data from youth football with women in Portugal would be, at the time of data collection, quite tricky, as the sporting conditions are different from male sport (e.g., it is pretty common to find 13 and 14-year old athletes playing for U19 teams and, therefore, the competitive level could be challenging to separate). Concerning this variable of competitive level, the study showed that even though the overall model was a good fit for both levels (U17 and U19), there are some nuances based on this variable. However, it was impossible to conclude that older athletes increase their expertise in dealing with stressful situations. Thus, it would be necessary for future studies to expand the range of ages being analyzed or follow athletes over time.

## Implications to practice and conclusion

The results have relevant implications for practice, particularly for sports psychologists. First, it highlights the importance of considering cognitive processes in stress adaptation, helping athletes generate challenging evaluations of the sporting events to better cope with them and experience more positive emotions. On the other hand, the study also emphasizes the need for stress management training because teaching athletes different strategies to cope with stressors may reduce their perceptions of threat when facing important stressful events. Specifically, goal-setting strategies can help athletes increase control over their performance (this was the major source of stress), and mental plans and imagery can help athletes deal better with competitive errors (the second major source of stress). Athletes can use these strategies before competitions by establishing specific and realistic goals and by defining positive thoughts to use when dealing with significant errors during competitions (Andersen, 2009; McArdle and Moore, 2012). Besides, arousal regulation, such as breath control and self-talk, can help athletes to control negative emotions and thoughts during and after competitions (Weinberg and Gould, 2019). These strategies can help athletes manage major stress sources and augment the positive pattern of adaptation to stressors (lower threat perception and higher challenge, control, and coping perceptions). In fact, there is evidence that having and training these mental skills is important for youth athletes. For example, the systematic review conducted by Dohme et al. (2019) identified the psychological skills that facilitate talented youth athletes’ development, as is the case of goal setting, social-support seeking, realistic self-evaluation, imagery, relaxation, maintaining a sense of balance, (pre)performance routines, and self-talk. The possess of these psychological skills by youth athletes augments their chances of overcome challenges and achieve athletic excellence (MacNamara and Collins, 2015).

Concluding, this study highlights the importance of considering how athletes perceive and cognitively evaluate competitive stressors to understand their emotional experience and how they adapt to a stressful event. Specifically, it showed that athletes who perceive competitive stressors as more challenging (and less threatening) assume more control over the demands and demonstrate to have more resources (coping perception) to deal with them, leading to more positive (and less negative) emotions. Moreover, it was also shown that these effects have stronger or weaker effects based on athletes’ competitive level.

## Author’s note

This study was conducted in the Psychology Research Centre (CIPsi/UM) of the School of Psychology, University of Minho and in the Research Centre for Human Development of the Faculty of Education and Psychology, Universidade Católica Portuguesa.

## Data availability statement

The raw data supporting the conclusions of this article will be made available by the authors, without undue reservation.

## Ethics statement

The studies involving human participants were reviewed and approved by Ethics Committee, School of Psychology, University of Minho. Ethics approval reference: SECSH-0162015. Written informed consent to participate in this study was provided by the participants’ legal guardian/next of kin.

## Author contributions

ARG was responsible for conceptualization, methodology, resources, writing – review and editing, and supervision. CS was responsible for methodology, formal analysis, and writing – review and editing. CM helped with the formal analysis and was responsible for writing – original draft. RC was responsible for investigation and helped writing the original draft. All authors contributed to the article and approved the submitted version.
